# Immuno-histochemical localization of endothelial nitric oxide synthase in testicular cells of men with non-obstructive azoospermia

**Published:** 2011

**Authors:** Khadijeh Foghi, Marefat Ghaffari Novin, Zahra Madjd Jabbari, Tohid Najafi, Mohammad Hasan Heidari, Abouzar Rostampour Yasoori

**Affiliations:** 1 **Infertility and Reproductive Health Research Center, **Shahid Beheshti University of Medical Sciences, Tehran, Iran.; 2 **Department of Biology and Anatomical Sciences, Faculty of Medicine, Shahid Beheshti University of Medical Sciences (M.C.), Tehran, Iran.**; 3 **Cancer Research Center, Iran University of Medical Sciences, Tehran, Iran.**

**Keywords:** *eNOS*, *Non obstructive azoospermia*, *Immunohistochemistry*, *Spermatogenesis*, *Human testicular tissue*

## Abstract

**Background:**

Non obstructive azoospermia (NOA) is one of the causes of male infertility in which spermatogenesis process is disturbed. Recent studies suggested the possible role of endothelial nitric oxide synthase (eNOS) in spermatogenesis process.

**Objective:**

The aim of the present study is to evaluate the expression of eNOS in human testicular tissue in men with NOA and men with normal spermatogenesis by using immunocytochemistry.

**Materials and Methods:**

In this case-control study, testicular biopsies were obtained from 10 men with NOA and 7 men with normospermia who were attended to infertility center for diagnosis or infertility treatment. Immunohistochemistry was used to localize the isoform of eNOS in these tissues and the intensity of staining was semi quantitively assessed. In addition, the histopathological evaluation was examined in both groups.

**Results:**

The isoform of eNOS enzyme activity was detected in the cytoplasm of sertoli and leydig cells in both groups. There was, however, a considerable variability in the intensity of staining between two groups. The expression of eNOS in Leydig cells in control group was significantly (p<0.05) higher than those in the NOA group. In contrast, expression of eNOS in Sertoli cells in NOA was more than those in the control group. eNO Simmune staining was absent in the normal germ cells but was intense in the abnormal germ cells with piknotic neucleous. The most histopathological finding were hypospermatogenesis (27.2%), Sertoli cell only syndrome (18.1%) and tubular fibrotic (13.6%).

**Conclusion:**

These results suggested that increase level of eNOS may play an important role in the apoptosis process in the abnormal germ cells and disturbance of spermatogenesis process.

## Introduction

Infertility is an important medical and social problem in the world (1). Male factor infertility is detected in about 40% of infertile couples (2). Azoospermia is defined as the absence of sperm in a centrifuged semen sample (3) and classified as either obstructive or non-obstructive. Obstructive azoospermia characterized by the testicular biopsy demonstrating sufficient spermatogenesis and physical occlusion of the reproductive tract distal to the testis that prevents sperm from entering the semen. Non-obstructive azoospermia is caused by severely reduced sperm production, resulting in the absence of sperm in semen (4). Indeed, non-obstructive azoospermia is primary testicular insufficiency due to some causes such as chromosome abnormalities, congenital, infections, malignancies, chemotherapy, heat, radiotherapy and such other unknown causes which result in abnormal spermatogenesis (5).

Recently, scientists have studied the role of nitric oxide in the spermatogenesis process (6). This molecule is made from L-arginine amino-acid when is converted into to L-citroline and nitric oxide, by the act of some co-factors and nitric oxide synthase enzyme (NOS) (7). 

Three isoforms of NOS including neuronal, endothelial and inducible have been identified and purified so far. Among these, endothelial isoform of nitric oxide synthase (eNOS) is depended to intracellular calcium (8, 9). Recent studies suggest that there are different physiological roles for nitric oxide in male reproductive system like incorporation in ejaculatory functions, androgen secretion, sperm motility, sperm capacitation, fusion of sperm with oocyte, etc. (10). 

By immune histochemical localization of eNOS, recent studies have suggested that eNOS expression has potentially important role in function of epididims, prostate, seminal vesicles as well as testicular tissues of humans and rats (11). eNOS has been localized in different cells in normal human testes such as endothelial cells, Leydig cells, Sertoli cells, and myo fibroblasts in seminiferous tubuls (7, 10). Meanwhile, there is not any study focusing on localization of eNOS in non- obstructive azoospermia in humans. Therefore, in this study, eNOS localization in testicular tissues of male with normal spermatogenesis and male with non-obstructive azoospermia with abnormal spermatogenesis has been studied. By comparing the expression of eNOS in both groups, it is possible to evaluate the role of eNOS in normal and abnormal spermatogenesis.

## Materials and methods

In this case-control study, immune histochemical evaluation were performed in testicular tissues of men with non-obstructive azoospermia (n=10) and men with normal spermatogenesis (n=7) who were attend to **infertility and reproductive health research center **for diagnosis or infertility treatment. Samples were obtained using testicular biopsy (TESE) under local anesthesia. Mean age and standard deviation of control and non– obstructive group were 30±1.5 and 34±1.5 years, respectively.All techniques and methods of biopsy and selection of patients were approved by ethical committee of Shahid Beheshti Medical University.

**Immunohistochemistry**

For immunehistochemical localization, testicular samples were fixed in Bouin’s fixative solution and after 4 hours were fixed again in 10% formalin solution. Then tissues were embedded in paraffin and later cut into 5 µm sections and placed on poly-L-Lysin coated slides.

Firstly, slides have been deparafinized, the endogenous peroxidase have been blocked by 0.3 % hydrogen peroxidase and absolute methanol (Merck Germany) for 15 minutes. Then non-specific antibody bindings have been blocked by goat serum (DAKO Denmark). Then, endogenous biotin has been arrested by means of blocking biotin solution for 15 minutes. Sections were incubated with Rabbit anti humaneNOS polyclonal primary antibody (cat num≠5589) for 1 hour at 37ºC (dilution:1:50). After washing, goat anti rabbit biotinated secondary antibody (Ab cam cat num≠6721) was added for 1 hour at 37ºC (dilution 1:200). After this step, third antibody has been treated for 30 minutes, with dilution of1:2000 (straptividin/HRP, Sigma, cat num≠2341). Then, 3, 3’ diaminobenzidine (DAKO cat num≠32000 Denmark) has been used as chromogen substance. Negative control samples were treated in the same way; but PBS has been replaced with primary antibody. Notably, for external positive control, the full term placenta tissue was used for histopathologic evaluation.

**Histopathology**

Testicular tissues were fixed in 10% formalin and embedded in paraffin. 5 µm thick sections were prepared and stained with hematoxylin and eosin and examined by a light microscope at 100 magnification using standard techniques. Testicular histology was classified into normal spermatogenesis, hypospermatogenesis (reduction in the degree of normal spermatogenic cells), sertoli cell only (absence of germ cells in seminiferous tubules) and tubosclerosis (no germ cells or Sertoli cells in the tubules).

**Statistical analysis**

Results of immune histochemical localization of two groups were evaluated with Kruskal-wallis non prarametric test. 

## Results

**Immunohistochemistry of eNOS**

The primary antibody used to detect eNOS was a commercially obtained polyclonal antibody and has been used and extensively characterized to confirm its specifity to the endothelial isoform of NOS, with use of the immune histochemical protocols described above. 

Tissues that incubated in PBS in the absence of primary antibody (negative control) showed no staining. An external control for eNOS staining consisted of human placenta in whicheNOS was consistently detected. Endothelial NOS was localized in testicular samples of control group and azoospermic patients group (Figure A). Our studies showed that (in both groups) special cells such as Leydig cells and sertoli cells, cytoplasm of premature spermatids and germ cells with picknoticneucleai- in apoptotic state- had eNOS staining. 

For the most part, germ cells were unstained. Leydig cells and Sertoli cells in non- obstructive azoospermia had higher staining rather than control group. Immature spermatids showed weak staining of eNOS in their cytoplasms. 

Germ cells with picnoticneuclei- like apoptotic neuclei and heterochromatin in non- obstructive azoospermia group had higher staining rate compared with two control groups. In addition, other cells such as endothelial cells of blood vessels and myoid muscular like cells showed some degrees of staining in immune histochemical assays.

**Histopathological findings**

The most histopathological finding were hypo-spermatogenesis (reduction in the degree of normal spermatogenic cells; 5 persons, 27.2%), Sertoli cell only syndrome (absence of germ cells in seminiferous tubules; 3 persons, 18.1%) and tubular fibrotic (no germ cells or Sertoli cells in the tubules; 2 persons, 13.6%).

**TableI:** Immunohistochemical localization of eNOS protein expression in human testicular cells in control and Non- obstructive azoospermic men

**Cell/ group**	**Sertoli cell**	**Leydig cell**	**Immature ** **spermatid**	**Normal germ cell**	**Abnormal germ cell** **( picknotic neuclei)**
Control group	+	++	+	0	+
NOA group	++	+	+	0	++

0: without staining; +: weak staining; ++: moderate staining and +++: power staining.

NOA: Non- Obstructive group.

**Figure 1:**
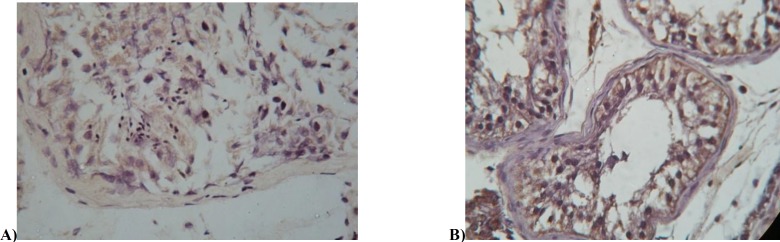
Immunostaining with eNOS antibody demonstrating staining in control group (A) and Non-obstructive azoospermic (B).

Testis section immune reacted with eNOS antibody showing staining in Sertoli cell (SC), Leydig cell (LC) (Arrow head) abnormal germ cell (AG).

## Discussion

Nitric oxide has important roles in different processes of different systems such as neuronal, immune and cardiovascular systems (12, 13).

Recently, nitric oxide production and eNOS expression has been identified in testicular tissue (14). eNOS enzyme is expressed in different cells of rat (13) and human (9) testes: Leydig, Sertoli, myofibroblasts, lamina propria around the seminifer tubules and degenerating germ cells (8). In the present study, eNOS enzyme expression in both groups and stain intensity in seminiferous tubules somatic cells (leydig, Sertoli, myofibrobalst, germ cells and immature spermatids) were analyzed. All cell types had different degrees of staining except normal germ cells that nearly did not have any staining. Zini *et al* (9) also localized eNOS to Leydig cells and Sertoli cells at all stage of spermatogenesis, in both histologically normal testes and testicular tissue of men with impaired spermatogenesis. Findings of immune histochemical assay of samples were in accordance with the findings of Zini *et al* in 1996 that performed eNOS immunohistochemistry on the testis and epididyms and vas deferens. One of the deficiencies of Zini’s studies was that their control group included patients with some spermatogenesis defects and also patients who had benign prostate hyperthrophy (BPH). In other words, control group didn’t have any normal testicular tissue. While, in this study, control group contains individuals with completely normal spermatogenesis and normal tissues that were biopsied to diagnose the reason of infertility (14). 

Naturally, in both groups there were abnormal populations of germ cells that were in the way of apoptosis. Since, there were vast spermatogenesis defects (from hypospermatogenesis to fibrotic tubules) in non-obstructive azoospermic group, naturally the percentage of abnormal germ cells in this group was high (8, 12). The process of normal aging is associated with high nitric oxide NO production and apoptosis in germ cells (12). In testes of older men, activity of inducible nitric oxide synthase iNOS and eNOS is elevated, so there are higher rates of apoptosis (13). 

Then with this hypothesis we can justify the higher staining intensity of eNOS enzyme in patients with non-obstructive azoospermia (10). eNOS and iNOS interfere in apoptosis of germ cells. Expression of eNOS is higher in germ cells related to other isoforms (11). Expression of eNOS enzyme in somatic cells (Leydig cells, Sertoli cells) and germ cells (immature spermatids and germ cells with picknotic nuclei) suggests that NO has potential role in spermatogenesis and it’s expression in abnormal germ cells and immature germ cells may be related to germ cells development (19). Recent studies showed that eNOS is involved in germinal epithelium function and finally in spermatogenesis (11). On the other hand, studies showed that proteins forming the blood testis barrier thigh junctions reacts structurally with eNOS enzyme, and finally results in opening or closing this junctions (12). NOS is critical regulator of permeability of epithelium. Researchers have shown that a NOS inhibitor (zinc protoporphyrin) (Zn pp) has considerable effects on dynamics of Sertoli cells (3) through NOS/ soluble guanylate cyclase and cyclic guanilate mono phosphatye (sGC/cGMP). 

Nitric oxide activates guanylatecyclase, result in activation of the signaling pathways-in thigh junction with Sertoli cells and then formation of cGMP that can disrupt tight junctions. cGMP activates G protein kinase too (15, 17, 18).While interaction between germ cells and Sertoli cells may regulate the expression of eNOS, it is not clear that which type of germ-cells is related to eNOS expression (8)

In conclusion, although there are different types of nitric oxide synthase in seminiferous epithelium, eNOS is the main regulator of spermatogenesis. This hypothesis can promote future studies to focus on prevention of azoospermic dependent abnormality and infertility. The over expression of eNOS in non-obstructive azoospermic men suggests that NO may play an important role in the apoptosis process in the abnormal germ cells and disturbance of spermatogenesis process.
